# MicroRNAs: exploring a new dimension in the pathogenesis of kidney cancer

**DOI:** 10.1186/1741-7015-8-65

**Published:** 2010-10-21

**Authors:** Nicole MA White, George M Yousef

**Affiliations:** 1 Department of Laboratory Medicine and the Keenan Research Centre in the Li Ka Shing Knowledge Institute, St Michael's Hospital, Toronto M5B 1W8, Canada; 2 Department of Laboratory Medicine and Pathobiology, University of Toronto, Toronto M5S 1A8, Canada

## Abstract

Renal cell carcinoma (RCC) is the most common neoplasm of the adult kidney. The role of the von-Hippel-Lindeau (VHL) tumour suppressor gene is well established in RCC with a loss of VHL protein leading to accumulated hypoxia-induced factor (HIF) and the subsequent transcriptional activation of multiple downstream targets. Recently, microRNAs (miRNAs) have been shown to be differentially expressed in RCC and their role in RCC pathogenesis is emerging. This month, in *BMC Medicine*, Gleadle and colleagues show that certain miRNAs are regulated by VHL in either a hypoxia-inducible factor (HIF)-dependent or HIF-independent manner in RCC. They also show that miRNA expression correlates with the survival of RCC patients.

In this commentary, we discuss the current understanding of the role of miRNAs in RCC and the different possible scenarios of their involvement in RCC pathogenesis. We also address their clinical significance as tumour markers, together with the potential use of miRNAs as therapeutic targets. Finally, we discuss some of the challenges that face the fast-evolving field of miRNAs, including the identification and validation of miRNA targets and the difficulties associated with establishing a link between miRNA expression and biological effects. A more thorough understanding of the biological nature of miRNAs and careful experimental planning will help us to reveal the complex role that miRNAs play in RCC pathogenesis.

See research article: http://www.biomedcentral.com/1741-7015/8/64

## Introduction

This month, in *BMC Medicine*, Gleadle and colleagues show, for the first time, the effect of the von-Hippel-Lindeau (VHL) tumour suppressor gene on microRNA (miRNA) expression in renal cell carcinoma (RCC) [1]. Their elegant work opens up a new dimension in the investigation of the role of miRNAs in the pathogenesis of RCC. It also highlights the potential challenges that need to be addressed in order to allow a better understanding of the complex relationship between miRNAs and cancer.

The VHL protein, which is defective in the majority of patients with RCC, plays a well characterized role in the pathogenesis of kidney cancer. Defective VHL leads to an accumulation of the hypoxia-inducible factor (HIF) and the activation of the hypoxic pathway of gene expression [2]. Anaerobic respiration seen in renal (and other) tumours can, therefore, be affected by the dysregulation of the VHL pathway. More recently, it has been shown that VHL has a number of other important functions that are HIF-independent [3]. The paper by Neal *et al. *identified a number of significantly dysregulated VHL-dependant miRNAs. Interestingly, whereas some of these miRNAs had altered expression as a consequence of HIF upregulation as previously documented in the literature, other miRNAs were dysregulated in a HIF-independent manner.

miRNAs are short non-coding, single-stranded RNAs that function by regulating protein translation and messenger RNA (mRNA) degradation of their target genes [4]. They have been shown to be involved in a number of critical biological processes, including cell development, differentiation and apoptosis [5,6]. It is estimated that about one-third of all human mRNAs are regulated by miRNAs [7]. Several independent studies have shown a strong link between dysregulated miRNAs and cancer, including breast, prostate, lung and kidney cancers [8-10]. Studies have shown that miRNAs affect the well-known pathways involved in tumorigenesis, including the regulation of cell cycle, proliferation, metastasis, angiogenesis and others [11].

## Discussion

**Figure 1:**
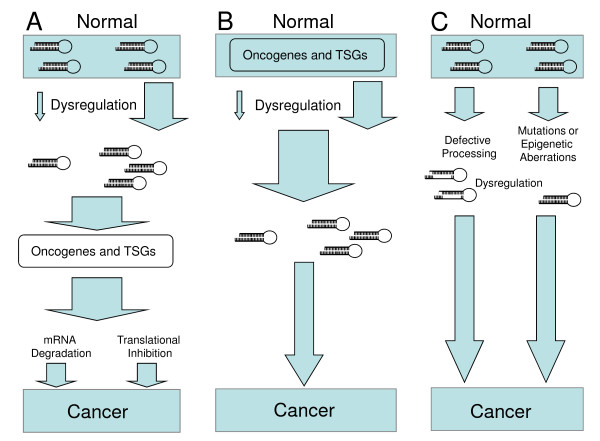
**The complex involvement of microRNAs (miRNAs) in tumourigenesis**. (A) Dysregulated miRNAs can have an oncogenic or tumour suppressor effect by inhibiting the protein production of their tumour suppressor or oncogenic targets, respectively. (B) Some miRNAs can be downstream targets of oncogenic of tumour suppressor genes, leading to their subsequent dysregulation and contribution to malignancy. (C) miRNA dysregulation can also be due to defective processing, mutations or epigenetic aberrations. TSG: tumour suppressor genes

As we proceed into more in-depth analyses of the role of miRNAs in cancer, it is now obvious that the relation of miRNAs with cancer is more complex than initially thought [11]. As shown in Figure 1, miRNA dysregulation can be either a cause or an effect of carcinogenesis. miRNAs can have oncogenic or tumour suppressor effects by inhibiting the protein production of their tumour suppressor or oncogenic targets, respectively. To add more complexity to this issue, some miRNAs have been shown to be downstream targets of oncogenic or tumour suppressor genes, such as p53 [12]. Moreover, it has been recently proposed that some miRNAs may be a part of the defence mechanism of the body's fight against carcinogenesis [13].

Evidence of the involvement of miRNAs in RCC pathogenesis is emerging. Recent studies have documented miRNA dysregulation in RCC compared to normal kidney tissue [10,14]. miRNAs can contribute to RCC pathogenesis at different levels. Some miRNAs have been shown to have an oncogenic effect on RCC [15]. The current study by Neal *et al. *showed that miRNA expression can be VHL-dependant.

It has been shown that several miRNAs are downstream effector molecules of the HIF-induced hypoxia response. miR-210 increases under hypoxic conditions and can target the iron-sulphur cluster protein (ISCU) which is involved in the mitochondrial electron transport chain, suggesting a potential mechanism for regulating anaerobic respiration in tumours [1,16,17]. Other miRNAs including miR-199a-5p and miR-135a [18] and miR-449a/b [19] have also been shown to be regulated by hypoxia.

Recent experiments have shown that, apart from the hypoxia pathway, miRNAs can directly affect tumour proliferation through mechanisms that are yet to be identified. As shown in the current study, miRNAs, including let-7i, miR-31, miR-21 and members of the miR-17-92 cluster, may also be involved in non-HIF mediated pathways in RCC [1]. The miR-17-92 cluster has been shown to act as an oncogene in many different cancers. Members of this cluster have been shown to be involved in the regulation of MYC-induced cell proliferation by targeting E2F1 expression. miRNAs can directly control VHL, HIF, vascular endothelial growth factor receptor (VEGFR) and other key molecules in RCC pathogenesis [8,10,11]. Lastly, the regulation of miRNA expression in RCC is complex, involving several mechanisms such as mutations, epigenetic changes, chromosomal aberrations and defective processing. We have recently shown that chromosomal aberrations in RCC (gain or loss) are responsible, at least in part, for controlling miRNA expression [8, 10, Youssef Y, White NM, Grigull J, Krizova A, Samy C, Mejia-Guerrero S, Evans A, Jewett M, Yousef GM: MiRNA profiling in kidney cancer subtypes: accurate molecular classification and correlation with cytogenetic and mRNA data identifies unique and shared biological pathway, Submitted].

On the clinical frontier, miRNA expression levels can serve as diagnostic, prognostic and predictive tumour markers [14,20-22]. In the study by Neal *et al*. [1], the expression of miR-210 was shown to be a prognostic marker as its expression correlated with patient survival. Other studies have also shown a correlation between other miRNAs and the different subtypes of RCC [23] and tumour size [10]. Previous studies in multiple cancers have provided the rationale of using miRNAs as potential cancer therapeutic targets [24-26]. This is also applicable to RCC, as recent reports have suggested that key molecules in kidney cancer pathogenesis represent potential targets of miRNAs [8,10]. miRNA gene therapy is particularly interesting because it offers the appeal of targeting multiple genes simultaneously. This is especially exciting as RCC is notorious for being resistant to therapy, especially in the metastatic stage.

The accompanying paper raises interesting technical challenges. The first of these is the identification and validation of miRNA targets. Currently, there are numerous target prediction programs but all of them lack sensitivity and specificity. The experimental validation of miRNA targets is an essential step towards a better understanding of the role of miRNAs in cancer pathogenesis. This is not, however, a simple process considering that target prediction is based on (1) partial complementarity with target mRNAs, (2) a single miRNA can target a large number of genes and (3) the fact that the same mRNA can be targeted by multiple miRNAs [21,27]. Establishing a high-throughput technique for the global validation of miRNA targets will need to be the corner-stone of miRNA research.

Although miRNA microarrays are a great initial tool for the discovery phase because they are operation-dependent their results are not consistent, they need to be validated by a gold standard technique (for example, the use of quantitative real time PCR with gene-specific primers) as shown in the paper by Neal *et al*. Also, since an individual miRNA can have multiple targets, special care must be taken in the experimental design and the appropriate controls must be included. These include the co-transfection of the miRNA and its antagonist (the antagomir) and a control, or 'scramble', miRNA. The latter can be somewhat problematical as, given the fact that miRNA interaction with the target relies on imperfect complementarity, it may give unexpected results. In this case, site-directed mutagenesis of the miRNA sequence may be preferred.

Another interesting challenge for miRNA research is the establishment of a direct relationship between miRNA dysregulation, target dysregulation and the end-point biological effect. This can be tricky as miRNAs can have an indirect effect on certain biological processes via alternate targets. Also, rather than being directly effected by the miRNA, the targeted gene may be a downstream target of these affected processes. Concurrent to developments in the miRNA field, improvements are required in animal models of diseases, in order to thoroughly test the effect of potential miRNA therapeutics.

## Conclusion

Current evidence shows that miRNAs will have a significant impact on our understanding of the pathogenesis of RCC. More studies are required in order to accurately identify the mechanisms by which miRNAs affect RCC. Moreover, miRNAs present new potential tumour biomarkers that will improve our diagnostic, prognostic and predictive abilities and, consequently, cancer patient management. Since the discovery that miRNAs can have direct biological effects on cancer, there has been much interest in developing novel miRNA-based cancer therapies. The development of a useful miRNA therapy has the capability to revolutionize personalized cancer therapy. Although many challenges exist in the field of miRNA research, continuing technological advances will help to overcome these challenges and enable us to understand the effect of miRNAs on RCC - and other tumour - pathogenesis.

## Abbreviations

HIF: hypoxia-induced factor; miRNA: microRNA; RCC: renal cell carcinoma; VHL: von-Hippel Lindeau.

## Competing interests

The authors declare that they have no competing interests.

## Authors' contributions

Both authors contributed equally to the development and writing of this manuscript.

## Pre-publication history

The pre-publication history for this paper can be accessed here:

http://www.biomedcentral.com/1741-7015/8/65/prepub
